# Retraction: MiR-132 enhances proliferation and migration of HaCaT cells by targeting TIMP3

**DOI:** 10.1039/d1ra90046c

**Published:** 2021-01-26

**Authors:** Laura Fisher

**Affiliations:** Royal Society of Chemistry Thomas Graham House, Science Park, Milton Road Cambridge CB4 0WF UK advances-rsc@rsc.org

## Abstract

Retraction of ‘MiR-132 enhances proliferation and migration of HaCaT cells by targeting TIMP3’ by Lina Jiang *et al.*, *RSC Adv.*, 2019, **9**, 21125–21133, DOI: 10.1039/C8RA10552A.

The Royal Society of Chemistry hereby wholly retracts this *RSC Advances* article due to concerns with the reliability of the data. The images in the article, and raw data provided by the authors, were screened by an image integrity expert. The expert concluded that the raw data provided by the authors was not genuine as western blot bands had been pasted onto a false background. In some instances it appeared that the bands in the article matched the raw data, but the backgrounds did not. Furthermore, the raw data provided by the authors was found to closely resemble raw data for a number of other articles, which is unexpected given that there are completely different authors lists for these articles.

Given the significance of the concerns about the validity of both the data in the article and the raw data provided by the authors, the findings presented in this paper are not reliable.

The authors have been informed but have not responded to any correspondence regarding the retraction.

Signed: Laura Fisher, Executive Editor, *RSC Advances*

Date: 15^th^ January 2021

## Supplementary Material

